# Flat-Band AC Transport in Nanowires

**DOI:** 10.3390/nano15010038

**Published:** 2024-12-29

**Authors:** Vicenta Sánchez, Chumin Wang

**Affiliations:** 1 Departamento de Física, Facultad de Ciencias, Universidad Nacional Autónoma de México, Mexico City 04510, Mexico; 2 Instituto de Investigaciones en Materiales, Universidad Nacional Autónoma de México, Mexico City 04510, Mexico

**Keywords:** flat band, AC conductivity, Kubo–Greenwood formula, independent channel method

## Abstract

The electronic states in flat bands possess zero group velocity and null charge mobility. Recently, flat electronic bands with fully localized states have been predicted in nanowires, when their hopping integrals between first, second, and third neighbors satisfy determined relationships. Experimentally, these relationships can only be closely achieved under external pressures. In this article, we study the alternating current (AC) in such nanowires having nearly flat electronic bands by means of a new independent channel method developed for the Kubo–Greenwood formula including hopping integrals up to third neighbors. The results reveal a large AC conductivity sensitive to the boundary conditions of measurement, where the charge carriers resonate with the external electric field by oscillating around their localized positions.

## 1. Introduction

In a flat electronic band, constituted by a huge number of compactly localized states resulting from the destructive interference, the charge carriers have an infinite effective mass and nil group velocity, which lead to a quenched kinetic energy and a zero conduc-ivity of direct current (DC). This highly degenerate energy level becomes a perfect platform to enhance strongly correlated electronic phenomena, such as the magic-angle-induced superconductivity observed in twisted bilayer graphene [1]. In the last decade, flat photonic bands have also been extensively studied, where unconventional light localization [2] and slow light propagation [3] are observed in engineered photonic lattices [4]. However, the alternating current (AC) in flat and nearly flat bands under low-frequency oscillating electric field is an important but less studied topic, in spite of the recent review of the universality of AC conduction in disordered solids [5] and a detailed study of electric conductivity at the zero-frequency limit in flat bands of the Su–Schrieffer–Heger model and Lieb lattice using two variations of the Kubo–Greenwood formula [6].

On the experimental side, the observation of flat bands requires fine-tuning around the critical conditions predicted by the theory, which have been nearly achieved leading to almost flat or extremely narrow bands [7]. In addition, nanowires with two-dimensional quantum confinement are currently performing a crucial building-block role in nanoelectronics [8]. In this article, we report a real-space tight-binding study of the AC conductivity carried out in cubically structured nanowires with nearly flat bands. A new independent channel method was developed for the Kubo–Greenwood formula including the first, second, and third neighbor hopping interactions, whose details can be found in Appendix A. This method combined with the previously developed real-space renormalization technique [9] allows an accurate calculation of the AC conductivity in mentioned nanowires of macroscopic length containing multiple structural interfaces commonly present in the AC measurement setup. When the flat-band or destructive-interference condition is closely satisfied, we observe the formation of several extremely narrow bands derived from the truly flat one, as well as an exceptionally high resonant AC conductivity at very low frequencies. These AC resonant peaks of a nanowire under external pressure can be used for the pressure measurement utilizing a simple electric circuit, instead of the widely used ruby fluorescence spectrum for pressure determination [10], since the external pressure modifies the electronic band width through hopping integrals and then the frequency of these resonant peaks.

## 2. Real Space Modeling

**Figure 1:**
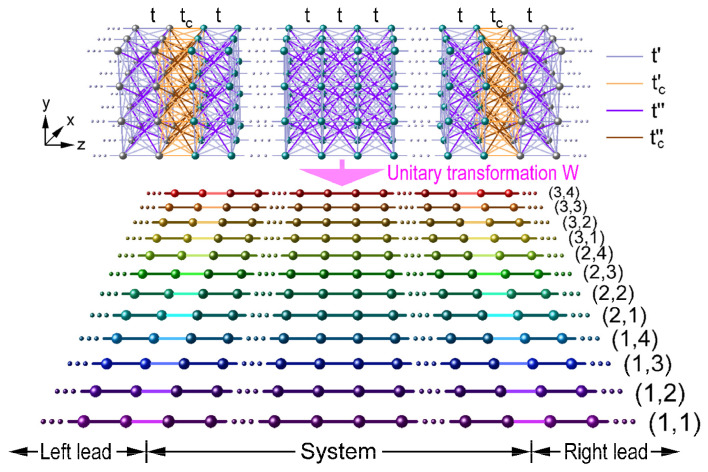
By means of a unitary transformation, a cubically structured nanowire with cross section of 3 × 4 atoms and hopping interactions up to third neighbors through $t,\;t^{\prime }$, and $t^{\prime \prime }$ is represented by 12 independent channels indexed by (α,β), where the system and its leads are connected by the hopping integrals $t_{c},\;{t^{\prime }}_{c}$ and ${t^{\prime \prime }}_{c}$ originated from their structural interfaces.

For the study of electronic transport in flat bands of a cubically structured nanowire, as well as the influence of AC measurement setup, we have chosen the real-space approach by means of a tight-binding model including hopping integrals between first (t), second ($t^{\prime }$), and third ($t^{\prime \prime }$) neighboring atoms, whose Hamiltonian (A6) through a unitary transformation $\hat{W}$ can be rewritten as a sum (A17) of those obtained from each independent channel, as discussed in Appendix A and schematically represented in Figure 1.

The Hamiltonian of channel (α,β) along the *Z*-directional can be expressed as

(1) $${\hat{H}}_{z}^{\left(\alpha ,\beta \right)}\;=\varepsilon (\alpha ,\beta )\sum_{k}\left|k\rangle \langle k\right|+\;\sum_{k}\left[t_{k}\;(\alpha ,\beta )\;\left|k\rangle \langle k\;+\;1\right|+t_{k-1}\;(\alpha ,\beta )\;\left|k\rangle \langle k\;-\;1\right|\right],$$

where *k* counts each atom of the channel, $\varepsilon (\alpha ,\beta )=E_{\alpha }\;+E_{\beta }\;+\;\;E_{\alpha }e_{\beta }$ is the on-site energy and $t_{k}\;(\alpha ,\beta )=(1+e_{\alpha }\;+e_{\beta }\;+e_{\alpha }e_{\beta })\;t_{k}$ is the nearest-neighbor hopping integral with $E_{\alpha }$, $E_{\beta }$, $e_{\alpha }$ and $e_{\beta }$ defined in (A12).

The channel (α,β) becomes to a fully disconnected chain with a highly degenerate flat band at energy equal to ε(α,β), if its hopping integrals became to zero, i.e.,

(2) $$1+e_{\alpha }\;+e_{\beta }\;+e_{\alpha }e_{\beta }\;=(1+e_{\alpha }\;)(1+e_{\beta }\;)=0.$$

In other words, conditions for the flat-band appearance are

(3) $$e_{\alpha }\;=2\tau \cos\;[\alpha \pi /\left(\;N_{x}\;+1\right)]=-1\;\mathrm{or}\;e_{\beta }\;=2\tau \cos\;[\beta \pi /\left(\;N_{y}\;+1\right)]=-1$$

for a nanowire containing $N=N_{x}\;\times N_{y}\;\times N_{z}$ atoms.

For example, let us consider a narrow nanowire of $3\times 4\times N_{z}$ atoms, which has a flat band at $\varepsilon (\alpha ,\beta )=\;E_{\alpha }\;+E_{\beta }\;+\;\;e_{\alpha }E_{\beta }\;=\;E_{\alpha }\;=\sqrt{2}\;|\;t\;|\;$, if α=3 in Equation (3), i.e., $t^{\prime }\;=\tau \;t=t\;/\sqrt{2}$ and $t^{\prime \prime }\;=\tau^{2}t=t\;/2$. On the other hand, if $t^{\prime }\;=t/\Phi$ and $t^{\prime \prime }\;=t/\Phi^{2}$ or β=4 in Equation (3), where $\Phi \;=\left(\sqrt{5}\;+1\right)/2$ is the golden ratio, the flat band would be located at $\varepsilon (\alpha ,\beta )=E_{\alpha }\;+E_{\beta }\;+\;\;E_{\alpha }e_{\beta }\;=E_{\beta }\;=\Phi \;|\;t\;|$. In the first case of $t^{\prime }\;=t\;/\sqrt{2}$, the degeneracy of flat band is $4N_{z}$, while it is $3N_{z}$ when $t^{\prime }\;=t/\Phi$, where $N_{z}$ is the number of atoms along the *Z* direction of nanowire.

**Figure 2:**
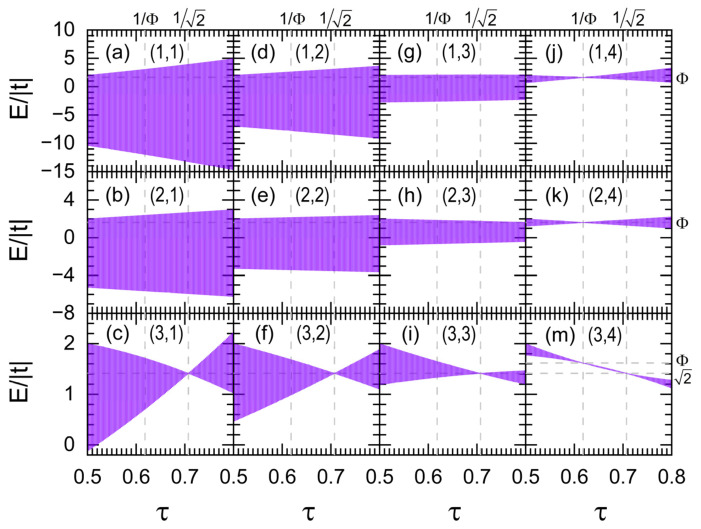
(**a**–**m**) Band width of independent channels (α,β), numbered in each figure, versus the hopping integral ratio $\tau \;=\;t^{\prime }/t$ for a nanowire with cross section of 3×4 atoms, being $\Phi \;=\left(\sqrt{5}\;+1\right)/2$.

In Figure 2, the band width of each independent channel (α,β) described by Hamiltonian (1) is plotted as a function of the ratio of hopping integrals $\tau \;=\;t^{\prime }/t$ for a nanowire of 3×4×11405774 atoms, which is connected at its ends to two semi-infinite periodic leads with the same cross section and Hamiltonian parameters of the system. Observe that channel (3,4), shown in Figure 2m, possesses two flat bands located at $E=\sqrt{2}\;|\;t\;|$ and E =Φ | t |. In contrast, channels (1,4) and (2,4) hold a single flat band at E =Φ | t |, while channels (3,1), (3,2), and (3,3) own the another at $E=\sqrt{2}\;|\;t\;|$.

On the other hand, the electronic density of states (*DOS*) can be calculated by means of the Green’s function $G(E)={\left(E-\hat{H}\right)}^{-1}$ determined by Hamiltonian (A6) as [11]

(4)  DOS(E)=−1πlimη→ 0+∑s=1NIm[Gs,s (E+iη)].

Using Equation (A30) in Appendix A, the *DOS* could be rewritten as

(5) $$\;DOS(E)=\sum_{\alpha =1}^{N_{x}}\sum_{\beta =1}^{N_{y}}DOS^{\left(\alpha ,\beta \right)}\;(E),$$

where

(6)  DOS(α,β) (E)=−1πlimη→ 0+Im[∑k=1NzGk,k(α,β) (E+iη)]=−1πlimη→ 0+Im∑k=1Nz〈k|1E+iη−H^z(α,β)|k〉

is the one-dimensional *DOS* of channel (α,β), which is efficiently calculated in this work by means of a real-space renormalization method given in Appendix A of ref. [9].

**Figure 3:**
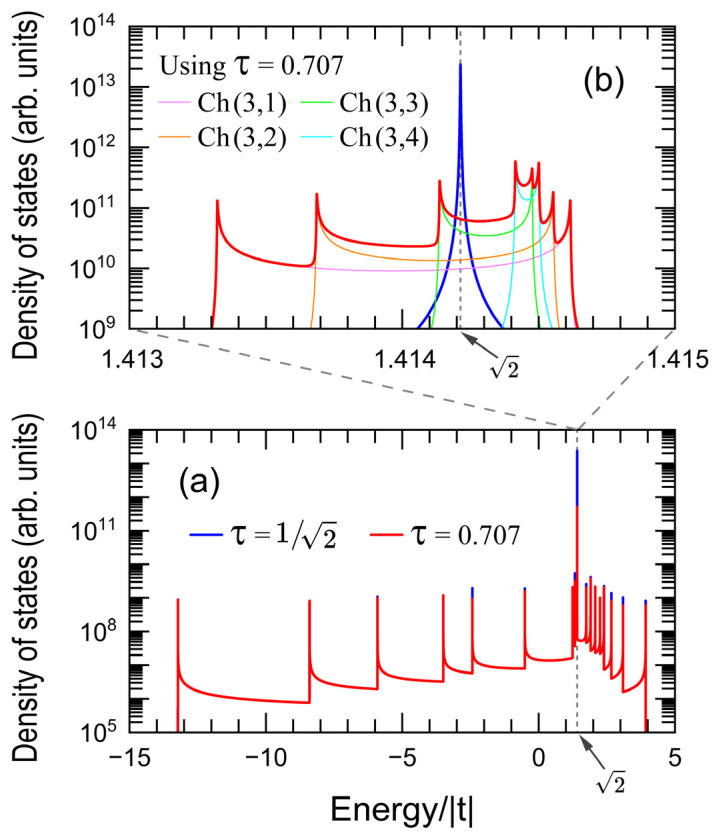
(**a**) Electronic density of states (DOS) versus energy with $\eta =10^{-6}\;|\;t\;|$ for the same nanowire of Figure 2 with $\tau \;=\;t^{\prime }/t=1/\sqrt{2}$ (blue line) and τ = 0.707 (red line). (**b**) Magnification of DOS around $E=\sqrt{2}|\;t\;|$ including those of channels (3,1) (magenta line), (3,2) (orange line), (3,3) (green line) and (3,4) (cyan line) for τ = 0.707.

Figure 3a shows the *DOS* in logarithmic scale as a function of the energy (*E*) for the same nanowire of Figure 2 with a hopping integral ratio $\tau \;=\;t^{\prime }/t=1/\sqrt{2}$ (blue line) and τ = 0.707 (red line), where an imaginary part of energy $\eta =10^{-6}\;|\;t\;|$ is used. Figure 3b presents a magnification of Figure 3a around $E=\sqrt{2}|\;t\;|$, where the *DOS* of channels (3,1) (magenta line), (3,2) (orange line), (3,3) (green line), and (3,4) (cyan line) using τ = 0.707 are also plotted.

Notice in Figure 3a the nearly flat band (red line) with τ = 0.707 possessing a *DOS* two orders of magnitude lower than that of the true one (blue line) at $E\;=\sqrt{2}\;|\;t\;|$ obtained from $\tau \;=1/\sqrt{2}$. Additionally, this almost flat band has a sophisticate band structure originated from the asymmetrical band broadening in channels (3,1), (3,2), (3,3) and (3,4) when τ = 0.707, as shown in Figure 3b, which may also be noted by comparing Figure 2c,f,i,m.

## 3. AC Conductivity

Within the linear response theory, the electrical conductivity (*σ*) can be calculated by means of the Kubo–Greenwood formula presented in Equation (A3), which could be analytically evaluated at zero temperature, for a periodic chain of *N* atoms connected to two semi-infinite periodic leads with the same parameters of the chain, leading to [9]

(7) $$\sigma (\mu ,\omega ,0)=\frac{8\;e^{2}t^{2}a}{\pi \;(\;N\;-1)\;\hbar^{3}\omega^{2}}\left[1-{\left(\frac{\mu -\varepsilon }{2t}\right)}^{2}\right]\left\{1-\cos\left[\frac{(\;N\;-1)\;\hbar \;\omega /\left(2t\right)}{\sqrt{1-\left[(\mu -\varepsilon \right)/(2t){]}^{2}}}\right]\right\}\;\Xi \left(2\;|\;t\;|-|\;\mu -\varepsilon \;|\;\right),$$

and its corresponding DC conductivity is [12]

(8) $$\sigma_{P}=\sigma (\mu ,0,0)=(\;N-1)\frac{e^{2}a}{\pi \hbar }\Xi \left(2\;|\;t\;|-|\;\mu -\varepsilon \;|\;\right),$$

where Ξ(x) is the Heaviside step function, *ε* is the on-site energy, *t* is the nearest neighbor hopping integral, and *a* is the interatomic distance in the periodic chain.

**Figure 4:**
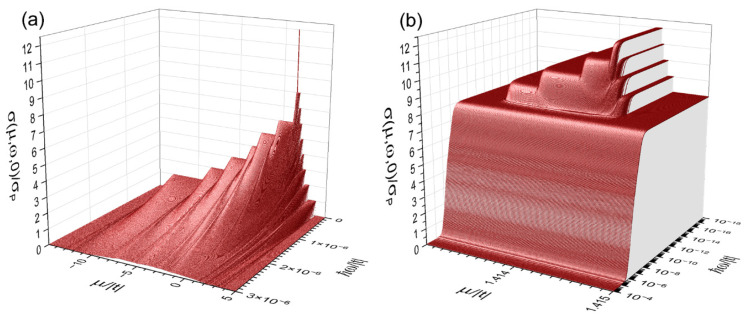
(**a**) Zero-temperature electrical conductivity $\sigma (\mu ,\omega ,0)/\sigma_{P}$ versus the chemical potential (μ) and the frequency (ω) for the same nanowire of Figure 3 with a hopping integral ratio $\tau \;=\;t^{\prime }/t=0.707$ and (**b**) its magnification around $\mu =\sqrt{2}|\;t\;|$ plotted in the logarithmic scale of frequency.

In Figure 4a, the AC electrical conductivity $\sigma (\mu ,\omega ,T)/\sigma_{P}$ at T =0 obtained from Equations (A36) and (7) is plotted as a function of the chemical potential (*μ*) and the external electrical-field frequency (ω) for the same nanowire of Figure 3 with a hopping integral ratio $\tau \;=\;t^{\prime }/t=0.707$. Notice the ballistic DC transport in each independent channel and the presence of nearly flat bands around $\mu =\sqrt{2}|\;t\;|$, as well as a general decreasing behavior of *σ* with the frequency in the interval $0\leq \hbar \;\omega \leq 3\times 10^{-6}\;|\;t\;|$. Figure 4b further illustrates an amplification of the $\sigma (\mu ,\omega ,0)/\sigma_{P}$ spectrum around $\mu =\sqrt{2}|\;t\;|$ using a logarithmic scale of frequency, where a ballistic DC conductivity is also observed in the four nearly flat bands, in contrast with the zero conductivity of truly flat bands when $\tau \;=1/\sqrt{2}$. However, such ballistic conductivity quickly vanishes when $\hbar \;\omega >10^{-10}\;|\;t\;|$ in parallel to its general disappearance in the rest bands starting from $\hbar \;\omega =10^{-7}\;|\;t\;|.$

The general decline of electrical conductivity around $\hbar \;\omega \approx 10^{-7}\;|\;t\;|$ can be noted from Equation (7), i.e., for an independent channel of N =11405774 atoms, when the chemical potential μ=ε and $\hbar \;\omega \ll 10^{-7}\;|\;t\;|$, we have $\cos\left[(\;N\;-1)\;\hbar \;\omega /\left(2t\right)\right]\approx 1-{\left[(\;N\;-1)\;\hbar \;\omega /\left(2t\right)\right]}^{2}$, which leads to an almost constant behavior of the conductivity, contrasted to the rapid decay with frequency, as much as $t^{2}/(\hbar \;\omega {)}^{2}$, when $\hbar \;\omega >10^{-7}\;|\;t\;|$. For the case of nearly flat bands, their hopping integral is about $10^{-4}\;|\;t\;|$, and then, the AC conductivity of these narrow bands starts their rapid decay around $\hbar \;\omega \approx 10^{-10}\;|\;t\;|$.

**Figure 5:**
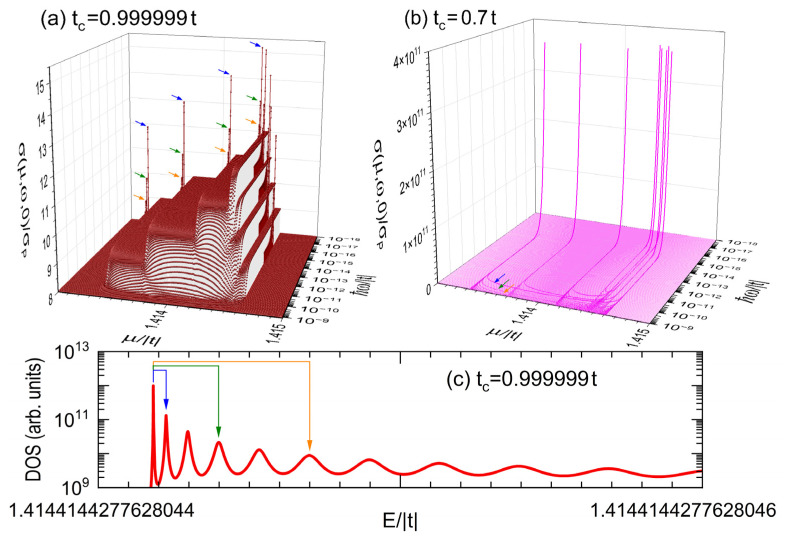
(**a**,**b**) Zero-temperature electrical conductivity σ(μ,ω,0) as a function of the chemical potential (*μ*) and frequency (ω) for the nanowire illustrated in Figure 1 with (**a**) $t_{c}\;=0.999999\;t$ and (**b**) $t_{c}\;=0.7\;t$. (**c**) Magnification of the density of states (DOS) as a function of energy (*E*) with $\eta =10^{-23}\;|\;t\;|$ around E=1.4144144277628045| t | for the case $t_{c}\;=0.999999\;t$.

Now, let us consider a more realistic measurement configuration of AC conductivity, where exist structural interfaces between the sample and metallic cables represented by periodic leads. These interfaces could be modeled by introducing new hopping integrals $t_{c}\;\neq t$, ${t^{\prime }}_{c}\;=\tau t_{c}$ and ${t^{\prime \prime }}_{c}\;=\tau^{2}t_{c}$, respectively denoted by camel, beige, and brown lines in Figure 1, that connect the first, second, and third neighboring atoms at the interface between the nanowire and its two semi-infinite periodic leads. In Figure 5a,b, the AC conductivity spectra around $\mu =\sqrt{2}|\;t\;|$ obtained from the Kubo–Greenwood formula (A3) using a small enough imaginary part of $\eta =10^{-27}\;|\;t\;|$ [13] are presented for the nanowire of Figure 2 with the connecting hopping integral (b) $t_{c}\;=0.999999\;t$ and (c) $t_{c}\;=0.7\;t$, while Figure 5c shows an amplification of *DOS* versus the electronic energy (*E*) with an imaginary part of $\eta =10^{-23}\;|\;t\;|$ in a small interval of [1.4144144277628044,1.4144144277628046]| t | for the case of $t_{c}\;=0.999999\;t$. It would be worth mentioning that all the numerical calculations presented in this article have been carried out in quadruple precision.

Observe the appearance of sharp resonant peaks at low frequency in Figure 5a, when a slightly perturbated connecting hopping integral of $t_{c}\;=0.999999\;t$ is introduced, where the presence of $t_{c}\;\neq t$ destroys the translational symmetry producing discrete energy spectra, as illustrated in Figure 5c and then, the presence of resonant AC conduction peaks [14]. Moreover, close to the largest resonant peak, indicated by blue arrows, there are several smaller ones at higher frequencies originated from electronic transitions, for example, between the ground state and the third (green arrows) or fifth (orange arrows) excited ones, in contrast with the forbidden transitions between the ground state and the second or forth excited ones, due to that the odd or even symmetry of wavefunctions plays a decisive role in the Fermi golden rule applied to the electric dipolar induced electronic transitions [15]. This resonant AC transport is even enhanced when $t_{c}\;=0.7\;t$ leading to maximal values of AC conductivity about $10^{11}$ times of the DC ballistic one ($\sigma_{P}$), as shown in Figure 5b. Observe also in Figure 5b the U-form location of AC resonant peaks in the μ-ω plane, which originated from the distribution of electronic states determined by the dispersion relation of finite periodic chains [16].

## 4. Conclusions

A new independent channel method for the Kubo–Greenwood formula, including hopping interactions between first, second, and third neighbors in cubically structured nanowires, is presented, which combined with the real-space renormalization method [9], permits a direct AC conductivity calculation of macroscopic length nanowires with multiple structural interfaces without additional approximations. Flat and nearly flat bands in such nanowires were analytically and numerically investigated, including their appearance conditions and the bandwidth of almost flat ones, where the destructive quantum interference conditions are strictly and approximately satisfied.

A narrow nanowire of 3×4×11405774 atoms with a ratio of the second-neighbor hopping integral ($t^{\prime }$) to the first-neighbor one (*t*), $\tau \;=\;t^{\prime }/t=0.707$, has been chosen as an example for the electronic transport study, where extremely high AC conductivity ($10^{11}$ times of the DC ballistic conductivity) is observed at a very low frequency, less than one Hz if $t_{c}\;=0.7\;t$ and | t | ≈1 eV. This resonant AC frequency sensitively depends on the value of τ and the system length, where the former determines the width of nearly flat bands, and the latter decides the distribution of electronic states in the energy scale.

In general, the flat band required a specific relationship between the first, second, and third hopping-integral strengths, which can be closely achieved by applying external pressures to the nanowire along X and Y directions, while the electronic band filling could be controlled via the application of a gate voltage. Despite the zero electronic mobility of flat bands, their charge carriers actually always oscillate around their localized positions, as suggested by the uncertainty principle of quantum mechanics. When these oscillations resonate with the frequency of the external electric field, a large AC response would be observed, as occurred in Figure 5b. As mentioned in the introduction section, these high resonant peaks can be used in the pressure measurement, since the experimental verification of nearly flat bands in nanowires is suggested to be carried out by applying hydrostatic external pressure to the cross-section of nanowires to achieve the flat-band appearance condition and the frequency of these resonant peaks is sensitive to the bandwidth through the ratio of hopping integrals.

Finally, it would be worth mentioning that the electron–electron and electron–phonon interactions are not explicitly included in the Hamiltonian of this study and their contributions within the mean-field approximation could be considered via hopping integrals depended on the electronic density and temperature. This extension of the study is currently being carried out. On the other hand, the combination of independent channel and real-space renormalization methods [17] presented in this article could be a useful tool in the design and study of aperiodic electronic and photonic devices containing multiple structural interfaces, like semiconductor diodes, bipolar junction and graphene transistors [18], as well as Fabry–Perot resonant cavities [19].

## Data Availability

Data are contained within the article.

## Appendix A. Independent Channel Method for the Kubo–Greenwood Formula

The electrical conductivity ($\sigma_{zz}$) of alternating current (AC) within the linear response approximation can be calculated by means of the Kubo–Greenwood formula given by [11],

(A1) σzz (μ,ω,T)=2e2ℏπ Ω m2∫−∞∞dEf(E)−f(E−ℏω)ℏωTr{p^zIm[G+ (E+ℏω)] p^zIm[G+ (E)] },

where the factor 2 counts the possible electron spin orientations, Ω is the volume of system, ${\hat{p}}_{z}$ is the projection of momentum operator along the *Z* direction or the external electric-field direction, and $f(E)={\left\{\exp[(E-\mu )/\left(k_{B}T\right)]+1\right\}}^{-1}$ is the Fermi–Dirac distribution with chemical potential μ and temperature *T*. The retarded $\left(G^{+}\right)$ and advanced $\left(G^{-}\right)$ single-electron Green’s functions are, respectively defined as [11],

(A2) $$G^{\pm }\;(E)\equiv \;\underset{\eta \;\to \;0^{+}}{\lim}G(E\pm i\eta )=\;\underset{\eta \;\to \;0^{+}}{\lim}\sum_{n}\frac{|n\rangle \langle n|}{E\pm i\eta -E_{n}},$$

where *η* is the imaginary part of energy and |n〉 is the eigenfunction of Hamiltonian satisfying the stationary Schrödinger equation $\hat{H}|n\rangle =E_{n}|n\rangle$. As Im(G+)=i (G− −G+ )/2, the Kubo–Greenwood formula (A1) can be rewritten in terms of the discontinuity $\left(\tilde{G}\equiv G^{+}\;-G^{-}\right)$ as

(A3) $$\sigma_{zz}\;\left(\mu ,\omega ,T\right)=-\frac{e^{2}\hbar }{2\pi \;\Omega \;\;m^{2}}\int_{-\infty }^{\infty }dE\frac{f(E)-f(E-\hbar \omega )}{\hbar \omega }Tr\left\{{\hat{p}}_{z}\tilde{G}(E+\hbar \omega ){\hat{p}}_{z}\tilde{G}(E)\;\right\},$$

whose trace could be expressed as [9]:

(A4) $$Tr\left\{{\hat{p}}_{z}\tilde{G}(E+\hbar \omega ){\hat{p}}_{z}\tilde{G}(E)\;\right\}=\;\underset{\eta \;\to 0^{+}}{\lim}\left\{S(E_{\omega }^{+},E^{+}\;)-S(E_{\omega }^{+},E^{-}\;)-S(E_{\omega }^{-},E^{+}\;)+S(E_{\omega }^{-},E^{-}\;)\right\},$$

where $E_{\omega }^{\pm }\;=E\pm i\eta +\hbar \omega$, $E^{\pm }=E\pm i\eta$ and using (A2) we have

(A5) $$S(E_{\omega }^{\upsilon },E^{\varsigma }\;)=Tr\left\{{\hat{p}}_{z}G(E_{\omega }^{\upsilon }){\hat{p}}_{z}G(E^{\varsigma }\;)\;\right\}$$

with υ and ς=+ or −.

For a cubically structured nanowire along the Z direction, containing $N_{x}\;\times N_{y}\;\times N_{z}$ atoms and transversal structural interfaces, the s-band tight-binding Hamiltonian of spinless electrons with null on-site energies and hopping integrals between first (t), second $\left(t^{\prime }\right)$ and third $\left(t^{\prime \prime }\right)$ neighbors is [20]

(A6) $$\hat{H}={\hat{H}}_{1}+{\hat{H}}_{2}+{\hat{H}}_{3},$$

where

(A7) $${\hat{H}}_{1}\;=\;\sum_{l,j,k}\left\{\;t\left|l,\;j,k\rangle \langle l\pm 1,\;j\;,k\right|+t\left|l,\;j,k\rangle \langle l,\;j\pm 1\;,k\right|+t_{k-1}\left|l,\;j,k\rangle \langle l,\;j,k\;-1\;\right|+t_{k}\left|l,\;j,k\rangle \langle l,\;j,k\;+1\;\right|\right\},$$

(A8) $$\begin{array}{c} {\hat{H}}_{2}=\sum_{l,j,k}\{\;t^{\prime }\left|l,j,k\rangle \langle l\pm 1,j\pm 1\;,k\right|+t^{\prime }\left|l,j,k\rangle \langle l\pm 1,j\mp 1\;,k\right|+{t^{\prime }}_{k-1}\left|l,j,k\rangle \langle l\pm 1,j\;,k-1\right|\;\\ +\;{t^{\prime }}_{k}\left|l,j,k\rangle \langle l\pm 1,j\;,k+1\right|+{t^{\prime }}_{k-1}\left|l,j,k\rangle \langle l,j\;\pm 1,k-1\right|+{t^{\prime }}_{k}\left|l,j,k\rangle \langle l,j\;\pm 1,k+1|\right\} \end{array},$$

and

(A9) $$\begin{array}{c} {\hat{H}}_{3}=\sum_{l,j,k}\{\;{t^{\prime \prime }}_{k-1}\left|l,j,k\rangle \langle l\pm 1,j\pm 1\;,k-1\right|+{t^{\prime \prime }}_{k-1}\left|l,j,k\rangle \langle l\pm 1,j\mp 1\;,k-1\right|\;\\ +\;{t^{\prime \prime }}_{k}\left|l,j,k\rangle \langle l\pm 1,j\pm 1\;,k+1\right|+{t^{\prime \prime }}_{k}\left|l,j,k\rangle \langle l\pm 1,j\mp 1\;,k+1\right|\} \end{array},$$

Respectively, describe the electron hopping between first, second, and third neighboring atoms. In Hamiltonians (A7), (A8), and (A9), *l*, *j* and *k* are integer numbers correspondingly counting atoms along the X, Y, and Z directions with wavefunctions in the Dirac notation as |l,j,k〉=|l〉|j〉|k〉 [21], being |l〉, |j〉 and |k〉 the one-dimensional (1D) Wannier functions. Hence, Hamiltonian (A6) can be rewritten as [22,23]

(A10) $$\hat{H}\;=\;{\hat{H}}_{x}\;⊗\;{\hat{I}}_{y}\;⊗\;{\hat{I}}_{z}\;+\;{\hat{I}}_{x}\;⊗\;{\hat{H}}_{y}\;⊗\;{\hat{I}}_{z}\;+\;{\hat{I}}_{x}\;⊗\;{\hat{I}}_{y}\;⊗\;{\hat{H}}_{z}\;+\;{\hat{H}}_{x}\;⊗\;{\hat{h}}_{y}\;⊗\;{\hat{I}}_{z}\;+\;{\hat{h}}_{x}\;⊗\;{\hat{I}}_{y}\;⊗\;{\hat{H}}_{z}\;+\;{\hat{I}}_{x}\;⊗\;{\hat{h}}_{y}\;⊗\;{\hat{H}}_{z}\;+\;{\hat{h}}_{x}\;⊗\;{\hat{h}}_{y}\;⊗\;{\hat{H}}_{z},$$

where symbol ⊗ denotes the Kronecker product, ${\hat{H}}_{z}\;=\;\sum_{k}\left(t_{k}\left|k\rangle \langle k\;+\;1\right|+t_{k-1}\left|k\rangle \langle k\;-\;1\right|\right)$, ${\hat{I}}_{\kappa }=\;\sum_{l}\left|l\rangle \langle l\;\right|$ for κ=x, y or z, ${\hat{H}}_{\lambda }\;=t\sum_{l}\left|l\rangle \langle l\;\pm \;1\right|$ and ${\hat{h}}_{\lambda }\;=\tau \sum_{l}\left|l\rangle \langle l\;\pm \;1\right|$ being λ=x or y, and τ is a dimensionless parameter with $t^{\prime }\;=t\;\tau$, $t^{\prime \prime }\;=t\;\tau^{2}$, ${t^{\prime }}_{k}\;=t_{k}\tau$ and ${t^{\prime \prime }}_{k}\;=t_{k}\tau^{2}$.

Now, we use the eigenstates |α,β〉=|α〉|β〉 of the nanowire’s cross-section on the XY plane to reduce Hamiltonian (A10) into a 1D effective one along the Z direction given by

(A11) $${\hat{H}}_{z}^{\left(\alpha ,\beta \right)}\;=\langle \alpha ,\beta |\hat{H}|\alpha ,\beta \rangle =E_{\alpha }{\hat{I}}_{z}\;+E_{\beta }{\hat{I}}_{z}\;+\;\;{\hat{H}}_{z}\;+E_{\alpha }e_{\beta }{\hat{I}}_{z}\;+e_{\alpha }\;{\hat{H}}_{z}\;+e_{\beta }\;{\hat{H}}_{z}\;+e_{\alpha }e_{\beta }{\hat{H}}_{z}$$

where $\alpha =1,\;2,\;\cdots ,N_{x}$, $\beta =1,\;2,\;\cdots ,N_{y}$, and [16],

(A12) $$\{\begin{array}{c} E_{\alpha }\;=\langle \alpha |{\hat{H}}_{x}\;|\alpha \rangle =2t\cos\;[\alpha \pi /\left(\;N_{x}\;+1\right)]\;\\ E_{\beta }\;=\langle \beta |{\hat{H}}_{y}\;|\beta \rangle =2t\cos\;[\beta \pi /\left(\;N_{y}\;+1\right)]\\ e_{\alpha }\;=\langle \alpha |{\hat{h}}_{x}\;|\alpha \rangle =2\tau \cos\;[\alpha \pi /\left(\;N_{x}\;+1\right)]\;\\ e_{\beta }\;=\langle \beta |{\hat{h}}_{y}\;|\beta \rangle =2\tau \cos\;[\beta \pi /\left(\;N_{y}\;+1\right)] \end{array}.$$

In fact, Hamiltonian (A11) can be rewritten as

(A13) $${\hat{H}}_{z}^{\left(\alpha ,\beta \right)}\;=\varepsilon (\alpha ,\beta )\sum_{k}\left|k\rangle \langle k\right|+\;\sum_{k}\left[t_{k}\;(\alpha ,\beta )\;\left|k\rangle \langle k\;+\;1\right|+t_{k-1}\;(\alpha ,\beta )\;\left|k\rangle \langle k\;-\;1\right|\right],$$

where $\varepsilon (\alpha ,\beta )=E_{\alpha }\;+E_{\beta }\;+\;\;E_{\alpha }e_{\beta }$ is the on-site energy and $t_{k}\;(\alpha ,\beta )=(1+e_{\alpha }\;+e_{\beta }\;+e_{\alpha }e_{\beta })\;t_{k}$ is the nearest-neighbor hopping integral of a Z-directional chain, denoted as (α,β) channel. In other words, the nanowire is transformed into a set of $N_{x}N_{y}$ independent channels by means of a unitary transformation $\left(\;\hat{W}\;={\hat{U}}_{x}\;⊗{\hat{U}}_{y}\;⊗{\hat{I}}_{z}\right)$ made of the eigenvectors of ${\hat{H}}_{x}\;⊗{\hat{H}}_{y}$, i.e.,

(A14) $$({\hat{U}}_{x}^{†}\;⊗{\hat{U}}_{y}^{†}\;)({\hat{H}}_{x}\;⊗\;{\hat{H}}_{y}\;)({\hat{U}}_{x}\;⊗{\hat{U}}_{y}\;)={\hat{U}}_{x}^{†}{\hat{H}}_{x}{\hat{U}}_{x}\;⊗{\hat{U}}_{y}^{†}{\hat{H}}_{y}{\hat{U}}_{y}\;={\hat{H}}_{x}^{diag}\;⊗\;{\hat{H}}_{y}^{diag},$$

where ${\hat{H}}_{x}^{diag}$ and ${\hat{H}}_{y}^{diag}$ are, respectively the diagonal version of matrices ${\hat{H}}_{x}$ and ${\hat{H}}_{y}$. In Equation (A14), we have used the following identity

(A15) (A⊗B)(C⊗D)=AC⊗BD.

Hence, Hamiltonian (A10) is transformed as

(A16) $$\begin{array}{ll} {\hat{W}}^{†}\hat{H}\hat{W} & ={\hat{H}}_{x}^{diag}\;⊗\;{\hat{I}}_{y}\;⊗\;{\hat{I}}_{z}\;+\;{\hat{I}}_{x}\;⊗\;{\hat{H}}_{y}^{diag}\;⊗\;{\hat{I}}_{z}\;+\;{\hat{I}}_{x}\;⊗\;{\hat{I}}_{y}\;⊗\;{\hat{H}}_{z}\;+\;{\hat{H}}_{x}^{diag}\;⊗\;{\hat{h}}_{y}^{diag}\;⊗\;{\hat{I}}_{z}\;\\ & +{\hat{h}}_{x}^{diag}\;⊗\;{\hat{I}}_{y}\;⊗\;{\hat{H}}_{z}\;+\;{\hat{I}}_{x}\;⊗\;{\hat{h}}_{y}^{diag}\;⊗\;{\hat{H}}_{z}\;+\;{\hat{h}}_{x}^{diag}\;⊗\;{\hat{h}}_{y}^{diag}\;⊗\;{\hat{H}}_{z} \end{array},$$

with ${\hat{H}}_{x}^{diag}\;=\;\sum_{\alpha }E_{\alpha }|\alpha \rangle \langle \alpha |$, ${\hat{H}}_{y}^{diag}\;=\;\sum_{\beta }E_{\beta }|\beta \rangle \langle \beta |$, ${\hat{h}}_{x}^{diag}\;=\;\sum_{\alpha }e_{\alpha }|\alpha \rangle \langle \alpha |$ and ${\hat{h}}_{y}^{diag}\;=\;\sum_{\beta }e_{\beta }|\beta \rangle \langle \beta |.$ Thus, given that ${\hat{I}}_{x}\;=\;\sum_{\alpha }|\alpha \rangle \langle \alpha |$, ${\hat{I}}_{y}\;=\;\sum_{\beta }|\beta \rangle \langle \beta |$ and ${\hat{I}}_{z}\;=\;\sum_{k}|k\rangle \langle k|$, (A16) can be rewritten as

(A17) $${\hat{W}}^{†}\hat{H}\hat{W}=\sum_{\alpha ,\beta }{\hat{H}}_{z}^{\left(\alpha ,\beta \right)}|\alpha ,\beta \rangle \langle \alpha ,\beta |\;,$$

which is consistent with Equation (A11).

In general, the projection of momentum operator along the Z direction may be calculated via [9]

(A18) $${\hat{p}}_{z}\;=\frac{im}{\hbar }[\hat{H},{\hat{z}}^{3D}],$$

where ${\hat{z}}^{3D}\;={\hat{I}}_{x}\;⊗{\hat{I}}_{y}\;⊗\hat{z}$ with $\hat{z}=\sum_{k}z_{k}|k\rangle \langle k|$, being $z_{k}$ the Z-direction Cartesian coordinate of atom *k*. Applying the same unitary transformation $\hat{W}\;$ to ${\hat{p}}_{z}$ and using (A17), we obtain

(A19) $${\hat{W}}^{†}{\hat{p}}_{z}\;\hat{W}\;=\frac{im}{\hbar }\left\{\left(\sum_{\alpha ,\beta }{\hat{H}}_{z}^{\left(\alpha ,\beta \right)}|\alpha ,\beta \rangle \langle \alpha ,\beta |\;\right)({\hat{I}}_{x}\;⊗{\hat{I}}_{y}\;⊗\hat{z})-({\hat{I}}_{x}\;⊗{\hat{I}}_{y}\;⊗\hat{z})\left(\sum_{\alpha ,\beta }{\hat{H}}_{z}^{\left(\alpha ,\beta \right)}|\alpha ,\beta \rangle \langle \alpha ,\beta |\;\right)\right\},$$

since ${\hat{W}}^{†}{\hat{z}}^{3D}\;\hat{W}\;={\hat{z}}^{3D}$. Employing (A13) and (A15), Equation (A19) can be written as

(A20) $${\hat{W}}^{†}{\hat{p}}_{z}\;\hat{W}=\frac{im}{\hbar }\sum_{\alpha ,\beta }|\alpha ,\beta \rangle \langle \alpha ,\beta |\left\{{\hat{H}}_{z}^{\left(\alpha ,\beta \right)}\hat{z}-\hat{z}\;{\hat{H}}_{z}^{\left(\alpha ,\beta \right)}\right\},$$

where

(A21) $$\begin{array}{ll} {\hat{H}}_{z}^{\left(\alpha ,\beta \right)}\hat{z} & =\sum_{k}\left[\varepsilon (\alpha ,\beta )|k\rangle \;\langle k|+t_{k}\;(\alpha ,\beta )|k\rangle \;\langle k+1|+t_{k-1}\;(\alpha ,\beta )|k\rangle \;\langle k-1|\right]\;\sum_{k^{\prime }}z_{k^{\prime }}|k^{\prime }\rangle \langle k^{\prime }|\\ & =\sum_{k}\left[\varepsilon (\alpha ,\beta )z_{k}|k\rangle \;\langle k|+t_{k}\;(\alpha ,\beta )z_{k+1}|k\rangle \;\langle k+1|+t_{k-1}\;(\alpha ,\beta )z_{k-1}|k\rangle \;\langle k-1|\right] \end{array}$$

and

(A22) $$\begin{array}{ll} \hat{z}\;{\hat{H}}_{z}^{\left(\alpha ,\beta \right)} & =\sum_{k^{\prime }}z_{k^{\prime }}|k^{\prime }\rangle \langle k^{\prime }|\sum_{k}\left[\varepsilon (\alpha ,\beta )|k\rangle \;\langle k|+t_{k}\;(\alpha ,\beta )|k\rangle \;\langle k+1|+t_{k-1}\;(\alpha ,\beta )|k\rangle \;\langle k-1|\right]\;\\ & =\sum_{k}\left[\varepsilon (\alpha ,\beta )z_{k}|k\rangle \;\langle k|+t_{k}\;(\alpha ,\beta )z_{k}|k\rangle \;\langle k+1|+t_{k-1}\;(\alpha ,\beta )z_{k}|k\rangle \;\langle k-1|\right]\;. \end{array}$$

In consequence, Equation (A20) converts to

(A23) $${\hat{W}}^{†}{\hat{p}}_{z}\;\hat{W}=\frac{ima}{\hbar }\sum_{\alpha ,\beta }|\alpha ,\beta \rangle \langle \alpha ,\beta |\left\{\sum_{k}\left[t_{k}\;(\alpha ,\beta )|k\rangle \;\langle k+1|-t_{k-1}\;(\alpha ,\beta )|k\rangle \;\langle k-1|\right]\right\},$$

where a uniform separation between atoms along Z direction, $a=z_{k}\;-z_{k-1}$, is assumed. Hence,

(A24) $${\hat{W}}^{†}{\hat{p}}_{z}\;\hat{W}=\sum_{\alpha ,\beta }{\hat{p}}_{z}^{\left(\alpha ,\beta \right)}|\alpha ,\beta \rangle \langle \alpha ,\beta |$$

with

(A25) $${\hat{p}}_{z}^{\;(\alpha ,\beta )}\;=\frac{ima}{\hbar }\sum_{k}\left[t_{k}\;(\alpha ,\beta )\;|k\rangle \;\langle k+1|-t_{k-1}\;(\alpha ,\beta )|k\rangle \;\langle k-1|\right].$$

On the other hand, the Green’s function is determined by the Dyson equation given by [11], $(E1-\hat{H})G=1$, which by applying the unitary transformation $\hat{W}\;$ and using (A17), we obtain

(A26) $${\hat{W}}^{†}\;\left[(E1-\hat{H})\;\hat{W}{\hat{W}}^{†}G\right]\hat{W}=\left\{E1-\sum_{\alpha ,\beta }{\hat{H}}_{z}^{\left(\alpha ,\beta \right)}|\alpha ,\beta \rangle \langle \alpha ,\beta |\right\}({\hat{W}}^{†}G\hat{W})=1$$

and then,

(A27) $${\hat{W}}^{†}G\hat{W}\;={\left\{E1-\;\sum_{\alpha^{\prime },\beta^{\prime }}{\hat{H}}_{z}^{\left(\alpha^{\prime },\beta^{\prime }\right)}\;|\alpha^{\prime },\beta^{\prime }\rangle \langle \alpha^{\prime },\beta^{\prime }|\right\}}^{-1}\;\sum_{\alpha ,\beta ,\gamma }|\alpha ,\beta ,\gamma \rangle \langle \alpha ,\beta ,\gamma |\;=\;\sum_{\alpha ,\beta }|\alpha ,\beta \rangle \langle \alpha ,\beta |\sum_{\gamma }\frac{|\gamma \rangle \langle \gamma |}{E\;-\;E_{\gamma }^{\left(\alpha ,\beta \right)}},$$

where ${\hat{H}}_{z}^{\left(\alpha ,\beta \right)}|\gamma \rangle =E_{\gamma }^{\left(\alpha ,\beta \right)}|\gamma \rangle$.

Given that the density of states (DOS) is related to the Green’s function as [11]

(A28) DOS(E)=−1πlimη→ 0+Im{Tr[G(E+iη)]}=−1πlimη→ 0+Im{Tr[W^†G(E+iη)W^]},

using (A27), $Tr\left\{\ldots \right\}=\sum_{u,v,w}\langle u,v,w|\ldots |u,v,w\rangle$ and |u,v,w〉=|u〉|v〉|w〉, (A28) could be rewritten as

(A29) DOS(E)=−1πlimη→ 0+Im{∑u,v,w〈u,v,w|∑α,β|α,β〉〈α,β|∑γ|γ〉〈γ|E +iη− Eγ(α,β)|u,v,w〉}={−1πlimη→ 0+Im[∑α,β∑w∑γ〈w|γ〉〈γ|w〉E +iη− Eγ(α,β)]},

and then

(A30)  DOS(E)=∑α,β{−1πlimη→ 0+Im[Tr(G(α,β)(E +iη))]}=∑α,βDOS(α,β) (E),

where $\;G^{\left(\alpha ,\beta \right)}(E\;+i\eta )=\sum_{\gamma }\frac{|\gamma \rangle \langle \gamma |}{E\;+i\eta -\;E_{\gamma }^{\left(\alpha ,\beta \right)}}$.

On the other hand, sum $S(E_{\omega }^{\upsilon },E^{\varsigma }\;)$ in (A5) can also be rewritten as

(A31) $$S(E_{\omega }^{\upsilon },E^{\varsigma }\;)=Tr\left\{{\hat{W}}^{†}{\hat{p}}_{z}\hat{W}{\hat{W}}^{†}G(E_{\omega }^{\upsilon })\hat{W}{\hat{W}}^{†}{\hat{p}}_{z}\hat{W}{\hat{W}}^{†}G(E^{\varsigma }\;)\hat{W}\;\right\}$$

where the identities $\hat{W}\;{\hat{W}}^{†}\;=1$ and Tr(ABC)=Tr(BCA) are used. Hence, using (A24), (A27), $Tr\left\{\ldots \right\}=\sum_{u,v,w}\langle u,v,w|\ldots |u,v,w\rangle$ and $\sum_{o,r,s}|o,r,s\rangle \langle o,r,s|=1$, Equation (A31) converts to

(A32) $$S(E_{\omega }^{\upsilon },E^{\varsigma }\;)=\;\sum_{u,v,w}\;\sum_{o,r,s}\;\sum_{l,h,k}\;\sum_{a,b,c}\left\{\begin{array}{l} \langle u,v,w|\left[\sum_{\alpha ,\beta }{\hat{p}}_{z}^{\left(\alpha ,\beta \right)}|\alpha ,\beta \rangle \langle \alpha ,\beta |\right]|o,r,s\rangle \langle o,r,s|\\ \left[\sum_{\alpha^{\prime },\beta^{\prime }}G^{\left(\alpha^{\prime },\beta^{\prime }\right)}(E_{\omega }^{\upsilon })|\alpha^{\prime },\beta^{\prime }\rangle \langle \alpha^{\prime },\beta^{\prime }|\right]|l,h,k\rangle \langle l,h,k|\\ \left[\sum_{\theta ,\phi }{\hat{p}}_{z}^{\left(\theta ,\phi \right)}|\theta ,\phi \rangle \langle \theta ,\phi |\right]|a,b,c\rangle \langle a,b,c|\\ \left[\sum_{\theta^{\prime },\phi^{\prime }}G^{\left(\theta^{\prime },\phi^{\prime }\right)}(E^{\varsigma })|\theta^{\prime },\phi^{\prime }\rangle \langle \theta^{\prime },\phi^{\prime }|\right]|u,v,w\rangle \end{array}\right\},$$

where $G^{\left(\alpha ,\beta \right)}(E_{\omega }^{\upsilon })=\;\sum_{\gamma }\frac{|\gamma \rangle \langle \gamma |}{E_{\omega }^{\upsilon }\;-\;E_{\gamma }^{\left(\alpha ,\beta \right)}}$. Given that, for example, |a,b,c〉=|a〉|b〉|c〉, $\sum_{a}|a\rangle \langle a|=1$ and $\langle h|b\rangle =\delta_{h,b}$, (A32) can be simplified to

(A33) $$S(E_{\omega }^{\upsilon },E^{\varsigma }\;)=\sum_{\alpha ,\beta }\;\sum_{w,s,k,c}{\hat{p}}_{w,s}^{\left(\alpha ,\beta \right)}\;G_{s,k}^{\left(\alpha ,\beta \right)}\;(E_{\omega }^{\upsilon }){\hat{p}}_{k,c}^{\left(\alpha ,\beta \right)}G_{c,w}^{\left(\alpha ,\beta \right)}\;(E^{\varsigma }),$$

which could be rewritten as

(A34) $$S(E_{\omega }^{\upsilon },E^{\varsigma }\;)=\sum_{\alpha ,\beta }Tr\left[{\hat{p}}_{z}^{\left(\alpha ,\beta \right)}\;{\tilde{G}}^{\left(\alpha ,\beta \right)}\;(E_{\omega }^{\upsilon }){\hat{p}}_{z}^{\left(\alpha ,\beta \right)}{\tilde{G}}^{\left(\alpha ,\beta \right)}\;(E^{\varsigma })\right].$$

Therefore, (A4) might be expressed as a sum of traces of each channel (α,β), i.e.,

(A35) $$Tr\left\{{\hat{p}}_{z}\tilde{G}(E+\hbar \omega ){\hat{p}}_{z}\tilde{G}(E)\;\right\}=\sum_{\alpha ,\beta }Tr\left[{\hat{p}}_{z}^{\left(\alpha ,\beta \right)}\;{\tilde{G}}^{\left(\alpha ,\beta \right)}\;(E_{\omega }^{\upsilon }){\hat{p}}_{z}^{\left(\alpha ,\beta \right)}{\tilde{G}}^{\left(\alpha ,\beta \right)}\;(E^{\varsigma })\right].$$

So, the Kubo–Greenwood formula (A3) in terms of independent channels can be written as

(A36) $$\sigma_{zz}\;\left(\mu ,\omega ,T\right)=\frac{1}{\Omega_{⊥}}\sum_{\alpha ,\beta }\sigma_{zz}^{\left(\alpha ,\beta \right)}\;\left(\mu ,\omega ,T\right),$$

where

(A37) $$\sigma_{zz}^{\left(\alpha ,\beta \right)}\;\left(\mu ,\omega ,T\right)=-\frac{e^{2}\hbar }{2\pi \Omega_{∥}m^{2}}\;\int_{-\infty }^{\infty }dE\frac{f\;(\;E)-\;f\;(\;E\;-\;\hbar \omega )}{\hbar \omega }Tr\left[{\hat{p}}_{z}^{\left(\alpha ,\beta \right)}\;{\tilde{G}}^{\left(\alpha ,\beta \right)}\;(\;E_{\omega }^{\upsilon }){\hat{p}}_{z}^{\left(\alpha ,\beta \right)}{\tilde{G}}^{\left(\alpha ,\beta \right)}\;(\;E^{\varsigma }\;)\right],$$

and $\Omega =\Omega_{∥}\;\Omega_{⊥}$, being $\Omega_{∥}$ and $\Omega_{⊥}$ correspondingly the volumes of system in the parallel and perpendicular subspaces with respect to the external electric field.
